# Fluoroscopic and Echocardiographic Illustrations of an Unsuccessful Locking of the GORE CARDIOFORM Septal Occluder Device

**DOI:** 10.1016/j.cjco.2020.07.020

**Published:** 2020-08-01

**Authors:** Stephane Noble, Hajo Müller

**Affiliations:** Department of Medicine, Cardiology Division, Structural Heart Unit, University Hospitals of Geneva, Geneva, Switzerland

A 59-year-old woman was referred for patent foramen ovale (PFO) closure for secondary prevention post paradoxical thrombo-embolic ischemic stroke. Transcranial Doppler and transthoracic echocardiography showed a large shunt during a Valsalva manoeuvre. Transesophageal echocardiography (TEE) confirmed a large PFO without aneurysm of the septum.

We implanted a 25-mm GORE CARDIOFORM Septal Occluder (W.L. Gore & Associates, Inc., Newark, DE) under general anesthesia. After the release of the PFO device, we noticed that the right disk was not properly locked (Fig. 1A). The loop of the locking system can be seen at the level of the middle marker (**arrow**), whereas it should be seen at the level of the marker of the right disk, as shown in a correctly deployed device (Fig. 1B). Furthermore, the distance between the right atrium marker and the middle marker should be < 10 mm (correct: Fig. 1B; incorrect: Fig. 1A). The final images of the procedural TEE showed that the right disk was expanded and pulsatile (Fig. 1C). At 6-month follow-up, the device had flattened and adopted its usual expected shape (Fig. 1D). There was no residual shunt at rest or during a Valsalva manoeuvre using microbubbles.

**Figure 1:**
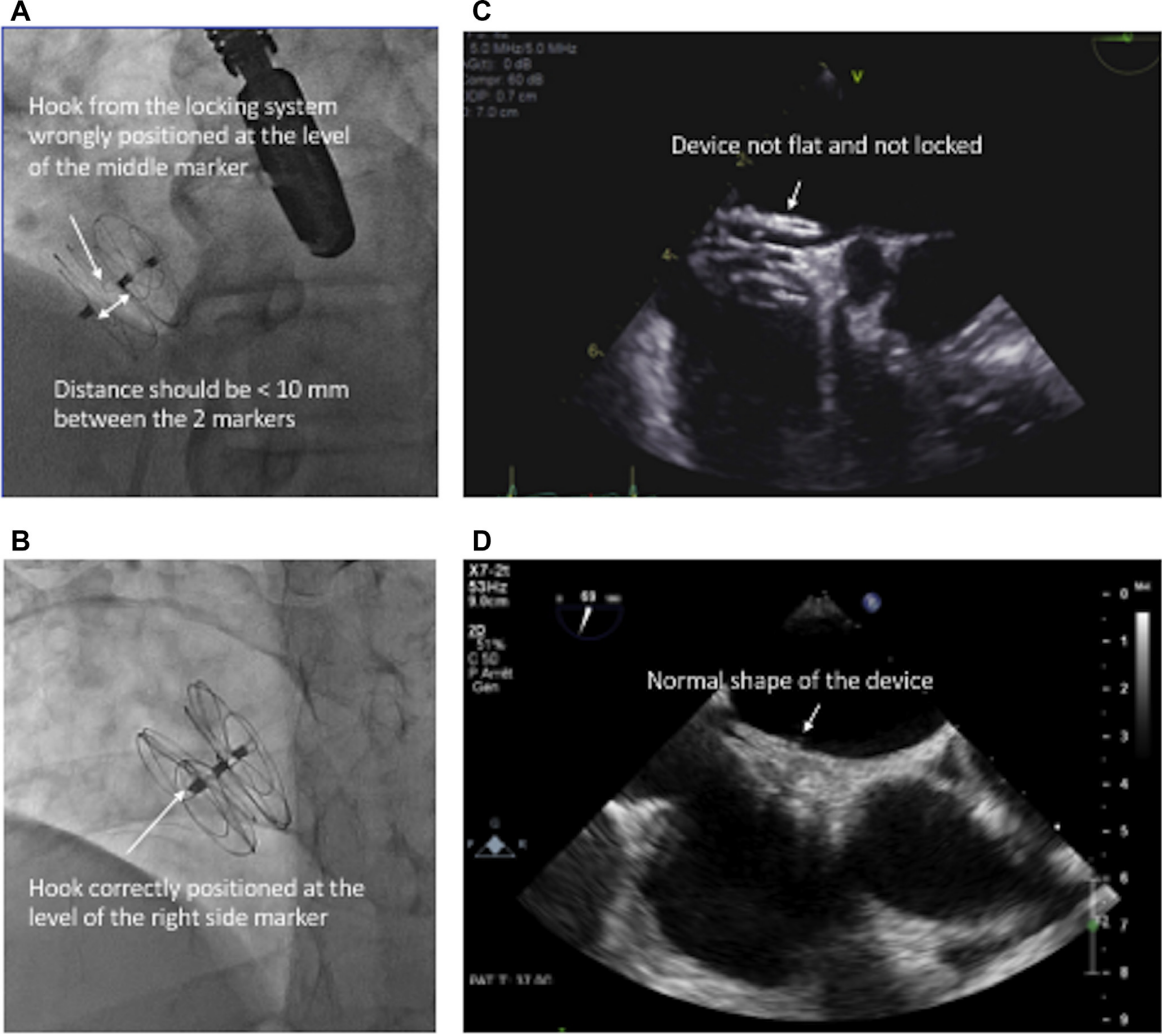
(**A**) After patent foramen ovale device release, the right disk is not properly locked. The loop of the locking system can be seen at the level of the middle marker (**arrow**), whereas it should be seen (**B**) at the level of the marker of the right disk, as shown in a correctly deployed device. Furthermore, the distance between the right atrium marker and the middle marker should be <10 mm ([**A**] incorrect; [**B**] correct). (**C**) Final images of the procedural transesophageal echocardiography showing that the right disk was not flat and therefore properly locked. (**D**) Transesophageal echocardiography at 6 months showing that the device flattened and took its usual expected shape.

This case nicely illustrates an unsuccessful locking of the system with an expanded right disk. We decided to leave the already totally released device in place as there was no residual shunt at rest but a moderate shunt during a simulated Valsalva manoeuvre. The alternative would have been to snare and retrieve the device. The 6-month check showed an excellent final result.

**Novel Teaching Points**

• On fluoroscopic images: the loop of the locking system should be seen at the level of the right disk marker; and the distance between the middle and the right disk marker should be < 10 mm.
• On TEE images: the device should be flat.

## Funding Sources

The 10.13039/501100006389University of Geneva (Geneva, Switzerland) provided funding for publication of this study.

## Disclosures

H. Müller and S. Noble received speaker honoraria from W.L. Gore & Associates, Inc. (Newark, DE).

